# Drug-induced hepatotoxicity among TB/HIV co-infected patients in a referral hospital, Ethiopia

**DOI:** 10.1186/s13104-019-4872-1

**Published:** 2020-01-02

**Authors:** Abebe Zeleke, Bethelihem Misiker, Teshager Aklilu Yesuf

**Affiliations:** Department of Pharmacy, Wollo University, Dessie, Ethiopia

**Keywords:** Tuberculosis, Human immunodeficiency virus, Hepatotoxicity

## Abstract

**Objectives:**

Anti-tuberculosis drug-induced hepatotoxicity is a common serious adverse drug reaction. This study intended to determine the prevalence and associated factors of drug-induced hepatotoxicity among tuberculosis and human immunodeficiency virus co-infected patients in Dessie referral hospital northeast Ethiopia.

**Results:**

In this cross-sectional study 84 patients were enrolled retrospectively. Data from September 1/2015 to August 30/2018 were extracted from March 1/2019 to April 1/2019. Association between dependent and independent variables was determined using the odds ratio and a *P* value of < 0.05 was considered as statistical significance. Out of 84 patients, 17 patients developed drug-induced hepatotoxicity which makes the prevalence of drug-induced hepatotoxicity 20.2%. The result revealed that the presence of disseminated or extrapulmonary tuberculosis [(AOR: 7.728, 95% CI (1.516–39.404)] and/or body mass index less than 18.5 kg/m^2^ [(AOR = 5.593, 95% CI (1.180–26.519)] were a risk factor for drug-induced hepatotoxicity. Tuberculosis and human immunodeficiency virus co-infected patients with extra- pulmonary tuberculosis and/or body mass index less than 18.5 kg/m2 should be closely followed and supervised for the development of hepatotoxicity.

## Introduction

Tuberculosis (TB) is one of the world’s deadliest communicable infectious disease particularly in developing counties where retroviral infection is rampant [1–5]. The 2010 world health organization (WHO) report ranked Ethiopia as the 7th country among the 22 high burden countries with tuberculosis and human immunodeficiency virus (HIV) co-infection and lower success rate of treatment [6].

Tuberculosis and HIV infections have epidemiological synergy where HIV patients have increased risk of developing active TB and higher rates of TB relapse and treatment failure [7].

The standard anti-TB regimen consisting of isoniazid, rifampicin, pyrazinamide and ethambutol given for 2 months followed by isoniazid and rifampicin for 4 to 7 months is effective but associated with adverse effects especially when taken simultaneously with anti-retroviral therapy [8–10]. Anti-TB drug-induced hepatotoxicity (ATDIH) is one of the most challenging clinical problems and the main cause of treatment interruption and associated with hospitalization and life-threatening events [11], which accounts for more than 7.0% of the overall adverse effects [3–5, 9].

Co-infection of TB with HIV is common and liver disease is becoming a leading cause of death which might be caused by HIV itself, hepatitis viruses, systemic opportunistic infections (OIs), malignancies, and drug-induced hepatotoxicity [6]. ATDIH is difficult to predict but evidences suggested that advanced age, excessive alcohol, preexisting chronic liver disease, chronic viral hepatitis B and C, HIV infection, advanced tuberculosis, malnutrition and concomitant administration of enzyme inducers increase the risk of ATDIH [7].

Determining patients with increased risk for ATDIH is essential to decrease cost of treatment, duration of illness, morbidity and mortality associated with drug induced hepatotoxicity [12]. In Ethiopia data regarding incidence and risk factors associated with ATDIH were limited and up to the investigators knowledge there was no study conducted in Dessie referral hospital. So, this study investigated the incidence of and determines risk factors for ATDIH in patients co-infected with TB/HIV.

## Main texts

### Methods

#### Study setting and study period

A cross-sectional study involving retrospectively enrolled TB/HIV co-infected inpatients and outpatients from September 1/2015 up to August 30/2018 were conducted in Dessie referral hospital from March 01–April 01, 2019. Dessie is the capital city of South Wollo zone, which is found 401 km north of Addis Ababa. The hospital delivers outpatient and inpatient services and has 15 specialists, 65 general practitioners, 195 B.Sc. nurses, 74 clinical nurses, 18 laboratory technicians and 23 lab technologists and 29 pharmacists and 12 pharmacy technicians. Generally the hospital has 535 health workers and 151 supportive staffs.

A total of 84 patients with TB/HIV co-infection who were treated from September 01, 2015 to August 30, 2018, who fulfilled the inclusion criteria were included in the study. TB/HIV infected patients who were on anti-TB regimen for at least 6 months and above, age greater than or equal to 18 years and normal and mild liver function tests (LFT) were included while patients with incomplete data and with liver toxicity were excluded from the study.

#### Study variables

Patient’s age and gender, CD4+ cell count, TB/HIV co-infection, WHO clinical stage of HIV/AIDS, type of TB, body mass index (BMI), type of antiretroviral therapy (ART) regimen, and history of OI- prophylaxis, viral load, or comorbid disease conditions and concomitant usage of co-medications were considered as independent variable and their effect were studied against the development of hepatotoxicity.

#### Data collection tools

Data were collected from patient medical charts using a data extraction tool which comprises; patient demographic and clinical data such as age, sex, and type of TB, CD4 + count, and ART status. The duration of treatment is retrieved from the ART clinic and TB registry.

#### Data processing and analysis

Data were edited, coded, entered to SPSS windows version 20.0.0. Descriptive statistics were used to determine the frequency and percentage. Prevalence of hepatotoxicity was calculated and the relationship between the dependent and independent variables was computed by logistic regression and Chi square tests (with a P-value of 0.05 and 95% CI). The processed data was compiled, organized and presented using tables, and figures.

#### Operational definitions

- Drug-induced Hepatotoxicity: the development of Hepatotoxicity due to anti- TB, ART and other concomitant use of drugs.
- Comorbidity; the presence of one or more diseases simultaneously with TB/HIV co-infection.
- Co-medication: concomitant use of medications other than Anti TB and HIV medications
- Hepatotoxicity; defined as a rise to ≥ threefold the upper limit normal (ULN) of alanine aminotransferase (ALT) and/or aspartate aminotransferase (AST).
- Mild hepatotoxicity: elevation of ALT/AST less than 3times ULN
- Moderate hepatotoxicity; elevations of ALT/AST from 3 to 5 times ULN
- Severe hepatotoxicity; elevations of ALT/AST from 5 to 10 times ULN
- Very severe hepatotoxicity; elevation of ALT/AST above 10 times ULN or elevations more than 250 IU/L with symptom of fulminant hepatitis as evidenced by jaundice and/or lethargy

### Results

#### Socio-demographic characteristics of participants

A total of 92 records of TB/HIV co-infected patients were reviewed and 84 meet the inclusion criteria and included in the study. From the total of 84, 52.4% of patients were males and the mean age of the patients was 41 years and ranges from 19 to 81 years. 58.3% of patients were in the BMI range 18.5–24.9 kg m^2^, and 14 patients had a history of alcohol intake and only 1 had a history of smoking (Table 1).

**Table 1:** Socio-demographic characteristics of TB/HIV co-infected patients from September 1/2015 to August 30/2018 (n = 84)

No.	Variables	Status	Number	Percent
1	Age	19–29	22	26.2
		30–39	25	29.8
		40–49	15	17.9
		Above 50	22	26.2
2	Sex	Male	44	52.4
		Female	40	47.6
3	BMI	Less than 18.5 kg m^2^	35	41.7
		18.5 up to 24.9 kg m^2^	49	58.3
4	Alcohol intake	Yes	14	16.7
		No	70	83.3
5	Smoking	Yes	1	1.2
		No	83	98.8
6	Education status	Illiterate	10	11.9
		Adult education	11	13.1
		Elementary school	14	16.7
		High school	25	29.8
		Diploma/degree	22	26.2
		M.Sc. and above	2	2.4
7	Marital status	Single	25	29.8
		Married	21	25
		Divorced	26	31
		Widowed	12	14.3
8	Occupation	Government employee	20	23.8
		Private employee	19	22.6
		Daily laborer	7	8.3
		Merchant	14	16.7
		Farmer	4	4.8
		Other	20	23.8

#### Clinical profiles of participants

From 84 study participants, the majority (66.7%) had PTB while the majority (66%) had a CD4 count less than 200. Study participants took four different types of ART regimens, the majority were on AZT/TDF + 3TC + EFV. Twenty-four participants had a comorbid condition in addition to TB/HV co-infection. From the total, 49 had a history of taking CPT prophylaxis. 20.2% had moderate to very severe hepatotoxicity, and only 6% had normal LFT values. An additional table file shows this (see Additional file 1).

#### Association between the development of hepatotoxicity and independent variables

To explore factors associated with increased risk of development of hepatotoxicity a bivariate and multivariate analysis were done. The bivariate analysis shows that BMI range, history of alcohol intake, Type of TB, WHO staging, history of OI-prophylaxis, adherence status, and viral load was significantly associated with the development of hepatotoxicity (Table 2).

**Table 2:** Factors associated with the development of hepatotoxicity (Bivariate analysis)

No	Variable	P value	OR	95% CI
1	BMI (18.6–24.9 kg m^2^)	–	1	Reference
	BMI (< 18.5 kg m^2^)	0.003	6.648	(1.941–22.771)
2	Alcohol history (no)	–	1	Reference
	Yes	0.028	4.023	(1.165–13.885
3	Type of TB (PTB)	0.011	1	Reference
	Extra pulmonary	0.019	4.487	(1.286–15.655)
	Disseminated	0.011	8.333	(1.642–42.283)
4	WHO clinical staging (3)	–	1	Reference
	Stage 4	0.002	5.844	(1.865–18.314)
5	OI- prophylaxis (no)	0.064	1	Reference
	CPT	0.401	2.537	(0.289–22.226)
	CPT and INH	0.066	8.0	(0.872–73.397)
6	Adherence (good)	0.027	1	Reference
	Fair	0.058	3.545	(0.958–13.128)
	Poor	0.009	8.125	(1.689–39.085)
7	Viral load (undetected)	–	1	Reference
	Detected	0.020	3.750	(1.226–11.468)

All variables that were found to have a P-value < 0.1 with hepatotoxicity in bivariate analysis were included in stepwise logistic regression (backward: likelihood ratio). Factors that were found to be associated with increased hepatotoxicity were those patients with BMI range less than 18.5 kg/m^2^ [(AOR = 5.593, 95% CI (1.180-26.519)], PTB as compared with extrapulmonary TB [(AOR: 7.728, 95% CI (1.516–39.404)] (Table 3).

**Table 3:** Factors associated with the development of hepatotoxicity (Multivariate analysis)

No	Variables	P value	AOR	95% CI
1	BMI range (18.6–24.9 kg m^2^)	–	1	Reference
	< 18.5 kg m^2^	0.030	5.593	(1.180–26.519)
2	Type of TB (PTB)	0.042	1	Reference
	Extra pulmonary	0.014	7.728	(1.516–39.404)
	Disseminated	0.195	4.070	(0.488–33.945)

### Discussion

This study focused on determination of the prevalence of drug-induced hepatotoxicity and associated factors among TB/HIV co-infected patients at Dessie referral hospital. A total of 84 patient records from September 01, 2015 up to August 30, 2018 were analyzed and the prevalence of developing hepatotoxicity was found to be 17 (20.2%). A comparable result was reported from Cameron (13.61%) [7]. Whereas a study from Brazil (36.7%) [5] and Ethiopia (30.0%) [4] reports a higher figure. However, the lower figure was reported from Jimma (11.5%) [3].

The effect of different factors such as age, sex, BMI, alcohol intake, type of TB, adherence, CD4+ count were assessed and lower BMI (≤ 18.5 kg/m^2^), and presence of extrapulmonary TB were associated with an increase in drug induced hepatotoxicity. BMI of less than 18.5 kg/m^2^ was mentioned as a factor in Jimma [3], whereas a study elsewhere did not found malnutrition as a factor [4, 13]. This significant association could be explained by derangement and disruption of drug metabolism pathways during protein-energy malnutrition including the acetylation pathways involved in isoniazid metabolism [7], possible depletion of glutathione stores, which makes patients more vulnerable to oxidative injuries, and the slower pace at which the liver metabolize drugs [3].

According to this study, the odds of developing hepatotoxicity were 7.7 times higher among patients with extrapulmonary TB as compared to patients with PTB. This was in congruence with studies in Cameroon and Ethiopia [3, 7]. The increased risk of developing hepatotoxicity in patients with extrapulmonary TB might be due to subclinical hepatic involvement, which plays a major role in developing drug-induced hepatotoxicity [3, 7].

Similar to a study from Brazil no significant associations were found between age and gender with the development of hepatotoxicity [5]. On the same token history of alcohol intake was not found to be significantly associated with hepatotoxicity. A similar finding was reported from Brazil and Jimma Ethiopia [3, 5], whereas a study from Loum District Hospital and Cameroon found a history of alcohol intake as one risk factor [7, 13]. This might be ascribable to the fact that the risk of hepatotoxicity depends on the amount of alcohol intake and in the current study the amount of alcohol intake was not studied. The current study didn’t find any significant association between concomitant use of drugs and the development of hepatotoxicity.

### Conclusions and recommendations

The prevalence of anti-tuberculosis drug-induced hepatotoxicity was high in Dessie referral hospital. From a total of 84 patients, the prevalence of developing drug-induced hepatotoxicity was 20.2%. BMI of less than 18.5 kg/m^2^, history of alcohol intake, type of TB, WHO stage 4, adherence status and viral load level was associated with hepatotoxicity. Patients with a BMI of less than 18.5 kg/m^2^ and patients with extrapulmonary TB had an increased risk of developing hepatotoxicity. TB/HIV co-infected patients presenting with extrapulmonary and BMI range lower than 18.5 kg/m^2^ or being malnourished need special attention due to the high risk they face. A more vigilant and regular monitoring of adverse effects, necessary organ function tests like liver function tests are mandatory while caring for these patient groups.

## Limitation of the study

This study had some limitations. Firstly, the study was conducted in a single hospital; therefore generalization of the finding must be made with caution. Secondly, the study used retrospective data of the liver function test and did not consider clinical findings of the patient; hence future studies should consider more than one liver function tests and the clinical features of the patient.

## Supplementary information

**Additional file 1.** Clinical parameters of TB/HIV co-infected patients from September 1/2015 up to August 30/2018 (n = 84).

## Data Availability

The datasets generated during the current study are available from the corresponding author on reasonable request.

## Abbreviations

ART: anti-retroviral therapy
ATDIH: anti-TB drug induced hepatotoxicity
BMI: body mass index
HIV: human immunodeficiency virus
LFT: liver function test
OI: opportunistic infections
PTB: pulmonary tuberculosis
TB: tuberculosis
WHO: World Health Organization
