# Supplementary material for: Drug-induced hepatotoxicity among TB/HIV co-infected patients in a referral hospital, Ethiopia
Source: BMC Res Notes. 2020 Jan 2;13:2. doi: 10.1186/s13104-019-4872-1 (PMC6941240; doi:10.1186/s13104-019-4872-1)
Supplement: Supplementary file 1 — Additional file 1. Clinical parameters of TB/HIV co-infected patients from September 1/2015 up to August 30/2018 (n = 84). [file 13104_2019_4872_MOESM1_ESM.pdf]

Additional file 1: Clinical parameters of TB/HIV co-infected patients from September 1/2015 up to August 30/2018 (n=84)

NO	Parameter	Status	Number	Percent
1	Type of TB	Pulmonary	56	66.7
		Extra pulmonary	20	23.8
		Disseminated	8	9.5
2	TB regimen	RHZE/RH	84	100
3	ART regimen	TDF + 3TC +EVF	38	45.2
		AZT + 3TC + EVF	29	34.5
		ABC + 3TC +EVF	12	14.3
		Others	5	6
4	Co-morbidity	Yes	24	28.6
		No	60	71.4
5	Co- medication	Yes	8	9.5
		N0	76	90.5
6	OI-prophylaxis	CPT	49	58.3
		CPT and INH	21	25
		No	14	16.7
7	Viral load	Less than 1000	51	60.7
		Above 1000	33	39.3
8	Adherence	Good	43	51.2
		Fair	30	35.7
		Poor	11	13.1
9	History of liver disease	No	79	94
		Yes	5	6
10	Liver Function Tests	<3 times ULN	67	79.76
		3-5 times ULN	14	16.67
		5-10 times ULN	2	2.38
		Above 10 times ULN	1	1.19
11	Development of hepatotoxicity	Yes	17	20.2
		No	67	79.8

ULN = upper limit of normal, RHZE/RH (R= Rifampicin, H=Isoniazid, Z =Pyrazinamide, E= Ethambutol), TDF = Tenofovir Disoproxil Fumarate, 3TC = Lamivudine, EFV= Efavirenz, AZT= Zidovudine, ABC = Abacavir, CPT = Cotrimoxazole preventive therapy, INH= Isoniazid
